# Functions and Implications of Autophagy in Colon Cancer

**DOI:** 10.3390/cells8111349

**Published:** 2019-10-30

**Authors:** Samantha N Devenport, Yatrik M Shah

**Affiliations:** 1 Cellular and Molecular Biology, University of Michigan, Ann Arbor, MI 48109, USA; 2 Molecular and Integrative Physiology, Internal Medicine, Division of Gastroenterology, and Rogel Cancer Center, University of Michigan, Ann Arbor, MI 48109, USA

**Keywords:** autophagy, nutrient, cancer, colon

## Abstract

Autophagy is an essential function to breakdown cellular proteins and organelles to recycle for new nutrient building blocks. In colorectal cancer, the importance of autophagy is becoming widely recognized as it demonstrates both pro- and anti-tumorigenic functions. In colon cancer, cell autonomous and non-autonomous roles for autophagy are essential in growth and progression. However, the mechanisms downstream of autophagy (to reduce or enhance tumor growth) are not well known. Additionally, the signals that activate and coordinate autophagy for tumor cell growth and survival are not clear. Here, we highlight the context- and cargo-dependent role of autophagy in proliferation, cell death, and cargo breakdown.

## 1. Introduction

Colorectal cancer (CRC) is the third leading cause of cancer-related deaths in the United States. It is estimated that in 2018, over 140,000 people were diagnosed with CRC [1]. Survival rates have improved in the last three decades due to early detection, however patients diagnosed at later stages of disease have a 5-year survival rate of 14% [2,3]. Treatments available for these patients are limited, therefore it is important to better understand tumor development and identify targeted therapies to improve overall patient care. Sporadic CRCs are typically marked by the initial loss of the adenomatous polyposis coli (*APC*) gene. APC is a scaffold protein that leads to proteasomal degradation of β-catenin. Under active cell states, WNT ligands bind to its receptor frizzled and prevent β-catenin degradation and activate target genes. Loss of APC constitutively activates β-catenin and causes uncontrolled epithelial proliferation. Mutations in *APC* are typically followed by sequential mutations in tumor protein *p53* (p53), and mutations in *KRAS* leading to spontaneous tumor development and progression [4]. It is well known that chronic inflammatory diseases such as Crohn’s Disease or ulcerative colitis increase risk of developing colon cancer, referred to as colitis-associated cancer (CAC) [5,6]. CACs developed from inflammatory bowel diseases (IBD) are a rare subset. Moreover, *p53* mutations occur earlier in the progression of CAC and *APC* mutations are less frequent and are found in late stage tumors in comparison to sporadic CRC [7]. However, CAC provides a clear link between inflammation and tumor initiation.

Autophagy is a highly regulated process that degrades and recycles cellular components. Dysregulation of autophagy is implicated in many diseases (as reviewed in [8]). Under several different cell stressors, autophagy is activated through kinase signaling and transcriptional activation by serine/threonine protein kinase 1 (ULK1) and transcription factor EB (TFEB). This activates a cascade of autophagy-related genes (ATG) [9], and formation of a spherical double layer membrane termed the autophagosome. The autophagosome delivers key cytoplasmic cargo such as organelles, foreign bodies, and cellular components to the lysosome for degradation into macromolecules that can be utilized by the cell. In CRC, autophagy is known to play tumor promoting and tumor suppressive roles [10,11], but the underlying mechanisms are not well understood. Studies have found conflicting functions of autophagy in tumors. These discrepancies are typically due to differences in the cells and tumor models that are utilized [12,13,14,15]. Further study of autophagy and its prevalence in CRC will uncover its potential therapeutic use [16]. Here we highlight cellular pathways that regulate autophagy, selective forms of autophagy, and how these mechanisms target different cargo for degradation.

## 2. Autophagy Subtypes

Autophagy can be classified into three major subtypes; macro-autophagy, micro-autophagy, and chaperone-mediated autophagy. There is a need for better understanding of cellular cues and the cell-dependent context by which autophagic subtypes are co-opted in cancer cells for growth and survival.

### 2.1. Chaperone-Mediated Autophagy

Chaperone-mediated autophagy (CMA) differs from macro-autophagy in that select proteins are targeted for degradation by direct targeting to the lysosome. Proteins are recognized by heat shock cognate protein 70 (HSC70). HSC70 interacts with lysosome-associated membrane protein type 2A (LAMP-2A) to internalize proteins into the lysosome. CMA substrates contain a specific motif, KFERQ, which is essential for HSC70 binding [17]. Relevant to cancer, inhibition of macro-autophagy enhances CMA-dependent degradation of mutant p53 [18]. Increased expression of LAMP-2A demonstrated activated CMA in CRC [19]. These functions highlight a potential role of CMA in tumor development. However, these processes have not been well studied in CRC.

### 2.2. Micro-Autophagy

**Figure 1:**
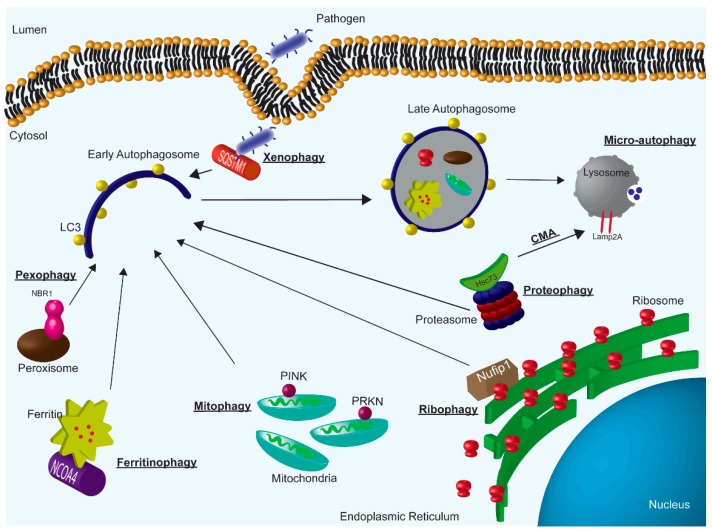
Overview of autophagy subtypes; macro-autophagy, micro-autophagy, and CMA. Specifically, highlighting examples of selective macro-autophagy.

Micro-autophagy is the direct engulfment of cellular components by invagination of the late endosome (Figure 1). Broadly, the implications of micro-autophagy have not been studied in many cancer types. There is evidence in lung cancer that amino acid starvation, an important factor in cancer growth (discussed below), induces micro-autophagy [20]. However, the role of micro-autophagy in CRC has not been investigated in detail.

### 2.3. Macro-Autophagy

Macro-autophagy can be broken down in to two subcategories that target cellular components for degradation; non-selective and selective. Non-selective macro-autophagy engulfs bulk cytosolic components and selective autophagy targets specific cargo for degradation (e.g., organelles and protein aggregates). For macro-autophagy, the phagophore, a precursor to the autophagosome, forms. Several ATG protein complexes are involved in early and late autophagosome formation [21]. As the membrane is forming, microtubule associated protein 1 light chain 3 beta (LC3-I) is conjugated with phosphatidylethanolamine and is processed into LC3-II [22]. Following fusion of the autophagosome with the lysosome, which contains the required enzymes for cargo degradation, LC3-II is broken down by the lysosome [23]. The turnover of LC3-II is often used as a readout of autophagic activity [24]. Here, we have provided a highly simplified overview of the complex process of macro-autophagy. This extremely coordinated event of cytoplasmic engulfment is generally activated in states of cell stress such as starvation. Anding and Baehrecke reviewed the important role of selective-autophagic processes in maintaining cellular homeostasis in response to stress [25]. Below we highlight the known selective-autophagic pathways and their potential role in CRC (Figure 1).

### 2.4. Mitophagy

Mitophagy is a process that selectively degrades mitochondria [26,27,28,29]. Mitophagy is highly evolutionarily conserved [30] and is known to be activated in yeast under starvation [31]. Therefore, highly proliferative cells under nutrient stress and starvation, such as cancer cells, may activate mitophagy. This process can be modulated by different pathways as discussed below.

#### 2.4.1. Parkin-Mediated Mitophagy

Under normal cell homeostasis, PTEN induced kinase 1 (PINK1) is maintained on the inner mitochondrial membrane. Upon damage or stress, PINK1 moves to the outer membrane and phosphorylates parkin (PRKN). This phosphorylation allows for ubiquitination of PRKN1 and targeting to the autophagosome. Poly-ubiquitination is recognized by adapters that direct mitochondria to the autophagosome. Known adapters include sequestosome (SQSTM1), neighbor of BRCA1 (NBR1), optineurin, nuclear domain 10 protein 52, and TAX1 binding protein; although, NBR1 is found to be non-essential for PRKN-mediated mitophagy [26,32,33]. Through this mechanism, a recent study found that in intestinal cancers, activation of mitophagy increased CD8+ T cells [34]. The upregulation of mitophagy causes an accumulation of iron followed by permeabilization of the lysosome. This permeabilization causes the release of proteases into the cytosol that induces presentation of MHC class I on the cell surface. This presentation elicits and an anti-tumor immune response by induction of CD8+ T-cells. In colitis, pharmacological induction of mitophagy through PRKN is found to inactivate inflammasomes in macrophages and ameliorate the impact of colitis [35,36,37]. While there are implications for PRKN-mediated mitophagy in CRC, one study found around 33% of colon tumors harbor PARK2 (the gene that encodes for PRKN) DNA copy number loss [38]. Interestingly, some colorectal cancer cell lines contain mutated forms of PRKN and may use alternate mechanisms to activate mitophagy [38]. Research is starting to investigate how PRKN may be used as a prognostic marker in CRC as it may correlate with invasion and overall survival [39].

#### 2.4.2. Parkin-Independent Mitophagy

Alternatively to PRKN directed mitophagy, interaction of LC3 to FUN14 domain containing 1 (FUNDC1) protein located on the outer mitochondrial membrane can also initiate mitophagy [40]. Another PRKN-independent mechanism includes BCL2 interacting protein 3 (BNIP3), a critical receptor for mitophagy [41]. In breast cancer, BNIP3 loss promoted tumor progression and metastasis [42]. BNIP3 is induced by hypoxia signaling, a critical micro-environmental stressor in CRC (discussed below) [43]. The impact of BNIP3 and mitophagy in CRC have not been studied in detail. The known function in breast cancer, and BNIP3’s relationship with hypoxia signaling provides a foundation to investigate the role in CRC. Mitochondria may also be recruited to the autophagosome by FKBP8, a member of the FK506-binding protein family. FKBP8 is located on the outer membrane and has an anti-apoptotic role by interacting with Bcl-2 [44]. PRKN-independent mitophagy can be initiated by binding of LC3A to FKBP8 [45]. Additional mechanisms of PRKN-independent mitochondrial control are reviewed by Stockum et al. [46]. PRKN-independent mechanisms, and the evidence of mutated PRKN discussed above in CRC highlight the potential importance of investigating PRKN- independent mitophagy in CRC.

Pharmacological targeting of mitochondria with Mito-CP or Mito-Met_10_ in *KRAS* mutant colorectal cancers induced mitophagy and decreased cell proliferation [47]. In colon cancer, treatment with a BH3 mimetic, which inhibits Bcl-2 anti-apoptotic proteins, induced mitophagy. Treatment with the mimetic, in combination with an mitophagy inhibitor, reduced CRC cell growth [48]. However, conflicting roles of mitophagy have been noted in cancer (Reviewed in [49]). Treatment with the mitophagy inhibitor liensinine increased breast cancer cell death [50]. Conversely, activation of mitophagy with ceramide, a molecule involved in sphingolipid metabolism, reduced tumor burden in acute myeloid leukemia [51]. In-depth understanding of mitophagy in CRC is needed in order to develop better therapies that can target mitophagy to reduce tumor growth.

### 2.5. Ribophagy

Ribophagy is the breakdown of ribosomes in cells, which constitute 10% of total cellular protein. Ribophagy is extremely low basally in cells [52]. Initiation of ribophagy occurs by the binding of nuclear FMR1 interacting protein 1 (NUFIP1) to ribosomes. This interaction leads to autophagosome recruitment by LC3 [53]. Starvation or molecular target of rapamycin complex 1 (mTORC1) inhibition induced NUFIP1 activity and increased ribophagy [53]. Breakdown of ribosomes under starvation underscores the importance of ribophagy for cellular nutrient maintenance. However, non-selective bulk degradation of ribosomes may also be utilized [52]. Little information is known about ribophagy in cancer. However, ribosomes contain a large amount of amino acids and nucleotides and can potentially serve as a nutrient store in the tumor environment.

### 2.6. Proteophagy

Clearance of proteasomes through autophagy is known as proteophagy. Cross-talk between the proteasome and autophagy is found under nitrogen starvation in cells wherein autophagy degrades ribosomes (and proteasomes) under nutrient starvation [54]. One of the earliest citations of proteophagy suggests that this process occurs through a chaperone-mediated mechanism where the proteasome is targeted to the lysosome by HSC73 [55]. More recent work uncovers the sequestration of the proteasome in autophagosomes under cell stress, suggesting proteophagy can occur in a macro-autophagy or CMA driven fashion [56]. Currently there is no data to suggest that proteophagy is activated in cancer.

### 2.7. Pexophagy

Peroxisomes are small organelle structures that break down fatty acids in the cytoplasm. The degradation of these products through autophagy requires SQSTM1 and NBR1 [57]. In healthy liver, loss of autophagy through ATG7 led to a buildup of peroxisomes [58]. Under starvation conditions, ubiquitination of peroxisomes occurred by peroxisomal biogenesis factor 2 (PEX2) in HeLa cells and mouse embryonic fibroblasts [59]. To our knowledge, the utilization of pexophagy in CRC has yet to be investigated. However, hypoxia inducible factor-2α (HIF-2α), an important transcription factor in CRC (discussed below), was found to promote pexophagy in hepatocytes [60]. While these findings were not investigated in colon tissue, the activation of pexophagy under starvation and hypoxia highlights the potential importance of studying pexophagy in CRC.

### 2.8. Ferritinophagy

Iron storage protein ferritin is broken down by the lysosome for iron release and cellular iron utilization. This degradation is directed by the nuclear receptor coactivator 4 (NCOA4) [61]. Interestingly, ferritinophagy is required for induction of ferroptosis, a form of cell death that requires iron [62,63]. Certain cancers have shown a sensitivity to ferroptosis [64] and pharmacological induction of ferroptosis is found to reduce pancreatic and hepatic cancer cell growth [63,65]. While little work has been done to investigate the importance of ferritinophagy in CRC, the essential role of iron in CRC growth [66] and the sensitivity of different cancer types to ferroptosis [67] highlights the importance in studying ferritin turnover in CRC.

### 2.9. Xenophagy

Xenophagy is a process initiated by the cell for protection against pathogens. Phagophores engulf pathogens and fuse to the autophagosome for breakdown by autophagy. Xenophagy can play a particularly important role in the colon due to the host-microbiome interaction. Protection from intestinal epithelial infection requires the autophagy gene *ATG16L1* [68]. Recent screening of xenophagy effectors identified a V-ATPase and ATG16L1 mechanism to specifically activate xenophagy under bacterial infiltration [69]. Certain bacteria can be targeted by SQSTM1, an important protein in autophagy [70]. In Crohn’s Disease, the stimulation of xenophagy using resveratrol reduced *Salmonella Typhimurium*, an enteric pathogen associated with Crohn’s Disease [71]. When colon cancer cells are treated with two mircoRNAs, MIR106B and MIR93, reduced ATG16L1 prevented removal of intracellular bacteria from epithelial cells via autophagy [72]. As mentioned, patients with Crohn’s Disease have an increased risk of developing CAC and this is partially due to bacterial infiltration. Understanding the role of xenophagy in host-microbiome homeostasis may be essential in characterizing the microbiota-tumor interaction.

## 3. Role of Autophagy in CRC

It is important to understand the role of autophagy at different stages and under different mutational loads to properly target tumors. A clinical study observed down-regulation of *ATG5* in CRC patients. However, increased expression correlated with increased incidence of invasion [73]. Conversely, expression of LC3B and SQSTM1 correlated with poor prognosis [74]. In mouse models, loss of *Atg7* in intestinal epithelial cells inhibited tumor growth through an immune response elicited by the microbiome [75]. Additionally, receptor for activated C kinase 1 (RACK1), a commonly found mutation in cancer, induced autophagy and promoted proliferation while inhibiting apoptosis in colon cancer [76]. Autophagy also modulated the degradation of the transcription factor FOXO3a in CRC. Inhibition of autophagy elevated levels of FOXO3a and led to transcriptional upregulation of pro-apoptotic genes [77]. Apoptosis also increased when autophagy was inhibited in CRC cells following activation of p53 and endoplasmic reticulum stress [78]. Conversely, treatment with Brevlin A increased autophagy and decreased tumor size [79]. This brief overview emphasizes the complexity of autophagy in CRC. It remains unclear if autophagy is anti- or pro- tumorigenic and in-depth mechanistic studies are needed. Table 1 outlines some of the opposing roles of autophagy in CRC.

An understanding of mutations in autophagy-associated genes, how autophagy is altered by increased mutational load, or via specific tumor suppressors or oncogenes is essential in assessing its role in tumor development. In CRC, there is low frequency of mutations in autophagy-associated genes [90]. In a small cohort of patient samples, tumors expressed decreased levels of ATG5, however increased expression correlated with invasion into lymphovascular tissue [73]. A study demonstrated that 95% of colon tumors expressed higher Beclin-1 compared to normal tissue [80]. These studies suggest that autophagy is important in cancer development. Similarly, in mutant *KRAS* cancers, autophagy induction occurred under starvation. Inhibition of *KRAS* reduced autophagy in these cells and inhibited cell growth [84]. In CRC-derived cell lines, p53 promoted the degradation of LC3 allowing for stable autophagic flux [81]. With loss of p53, LC3 accumulated and led to apoptosis. In CRCs with high microsatellite instability, 27% of the cancers harbored at least one mutation in either *ATG2B*, *ATG5*, *ATG9B*, or *ATG12* [91]. Additionally, a study aimed to understand responsiveness to therapy in BRAF (a protein involved in RAS/MAPK signaling) mutant colon cancers found that treatment with EGFR antibodies and checkpoint inhibitors induced autophagy and combining these treatments with an autophagy inhibitor reduced CRC cell growth [92]. Patients with Crohn’s Disease have an increased chance of developing CAC. Deficiencies in the response to bacterial sensing and invasion were observed following loss of autophagy through impairment in nucleotide-binding oligomerization domain (NOD1/2) signaling [93]. Loss of autophagy or mutations in autophagic genes may increase bacterial infiltration, which can impact the development of CAC. Mutations in autophagy-associated genes, or regulation of autophagy through mutations in genes such as *KRAS* and *p53*, demonstrate the important role of this mechanism in CRC. To begin addressing how autophagy can be used clinically, researchers have found a gene signature based on nine autophagy related genes that can accurately predict survival in colon cancer [94].

Histone deacetylase inhibitors as stand-alone or adjuvant therapies are currently used in several cancers [95]. In colorectal cancer cells, inhibition of autophagy through chloroquine, in combination with the histone deacetylase inhibitor vorinostat, led to an accumulation of ubiquitinated proteins and increased cell death [85]. Additionally, chemo-resistance required decreased autophagy in 5-fluorouracil (a common chemotherapeutic for CRC) resistant cells [86]. The authors speculated, that this observation was due to low autophagy resulting in accumulation of tumor promoting oxidative stress, inflammation, and damaged mitochondria.

CRC consist of multiple epithelial cell types as well as infiltrating immune cells. In IBD it is clear that dysregulation of autophagy in Paneth cells impacts tissue injury and inflammation [96]. However, cell type specificity of autophagy in tumor growth is unclear.

Immune cell autophagy: The anti-tumor immune response can directly kill cancer cells. As the tumor progresses, the microenvironment shifts to a highly immunosuppressive state and many of the immune cells potentiate tumor growth. Immunosuppression is essential in enhancing tumor progression, and immune cells can employ autophagy to perform standard functions including antigen presentation and cytokine production (Reviewed in [97]). In tumor-associated macrophages (TAM)s, upregulation of autophagy reduced tumor growth and increased apoptosis in CRC cells. Moreover, radiosensitization of CRC required increased autophagy in TAMs [98]. Conversely, when autophagy is lost in regulatory T-cells by disrupting *Atg7*, there was impaired ability of the anti-tumor immune response to CRC cells [99]. This was due in part by increased apoptosis in the T-cells. While not specifically studied in colon cancer, different immune cells including neutrophils, macrophages, B-cells, and natural killer cells rely on autophagy for their development and function (Reviewed in [100]). Immune cell specific autophagy underscore the importance of investigating this pathway in different cell types to better develop strategies for modulating tumor growth. 

**Figure 2:**
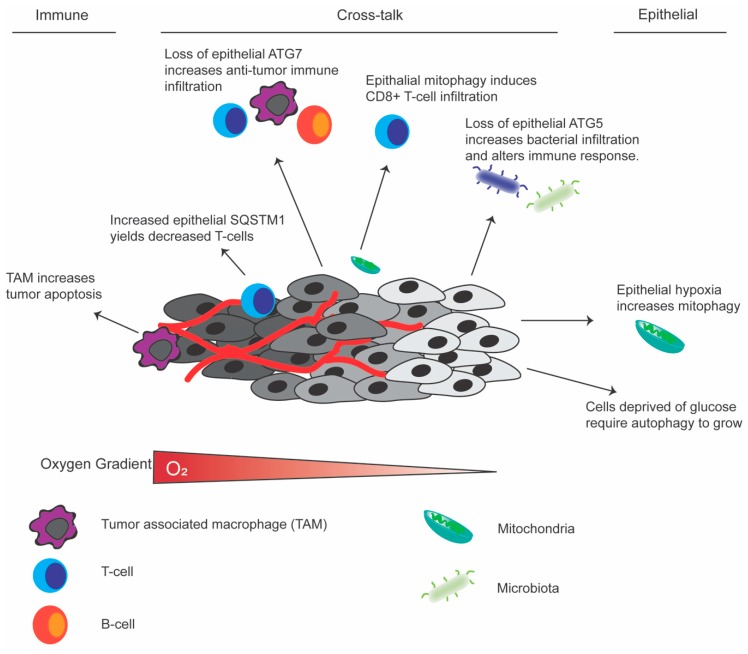
Schematic of the tumor microenvironment highlighting the impact of autophagy. Cell autonomous roles of autophagy in immune, epithelial or, the cross-talk between cell types in colorectal cancer.

Epithelial autophagy: Tumor epithelial autophagy in *KRAS*-driven cancers alters inflammatory mediators to suppress the immune response [82]. Furthermore, inhibition of autophagy in cancer cells blocked interferon gamma-mediated cell death [101]. The role of the immune system and its interaction with the gut microbiota is important in tumor development. Cell autonomous autophagy in healthy epithelial cells altered barrier function by breaking down junctional proteins such as claudin 2 [102]. Impaired barrier function can lead to increased bacterial infiltration to cause inflammation and damage in the gut. Recent work demonstrated tumor stage specific changes in bacterial infiltration, inflammatory signaling and cancer progression and growth in CRC [103]. This suggests a possible role of epithelial xenophagy in CRC. In CRC tumors, regulatory T-cell infiltration inversely correlated with SQSTM1 expression [104]. The utilization of autophagy in epithelial cells may alter recruitment or function of the immune response. In summary there are major differences in the direct impact of autophagy in epithelial cells, immune cells, or the heterocellular cross-talk between these cells that can impact CRC growth and progression (Figure 2). Understanding the changes in autophagy and how it impacts tumor response will allow researchers to further understand these mechanisms in different cell types.

## 4. Cellular Cues for Autophagic Activation in Cancer

### 4.1. Starvation

**Figure 3:**
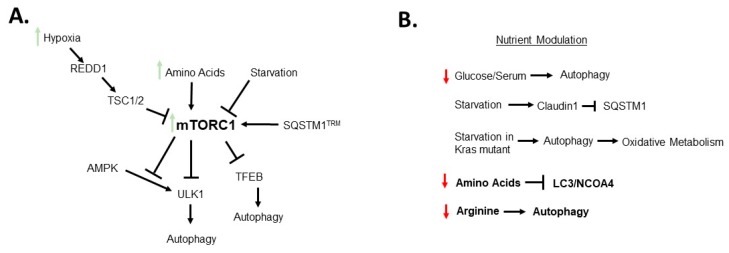
Schematic summarizing (**A**) Simplified overview of mechanisms of mTORC1 regulation. and (**B**) how nutrient modulation impacts autophagy. Bolded mechanisms indicate data from non-CRC samples. Please refer to text for detailed mechanisms.

The highly proliferative nature of tumors leads to a reduction in availability of nutrients in the microenvironment. In cancer, hyper-activation of mTORC1, a known pathway of nutrient sensing, contributes to cell proliferation and tumor progression. mTORC1 is activated in about 50% of CRC tumors. Figure 3A outlines the known mechanistic cross-talk in CRC between autophagy and mTORC1. In conditions where amino acids are abundant, mTORC1 is localized to the lysosomal membrane (Reviewed in [105]). Hypoxic induction of DNA damage inducible transcript 4 (REDD1) signaling has been shown to regulate mTORC1 through truncation of the hemartin (Tsc1/Tsc2) complex [106]. Since mTORC1 is activated by available nutrients, a feedback loop exists between these two mechanisms wherein autophagy generates new macromolecules to activate mTORC1. The cross-talk between these two mechanisms are essential in maintaining cell growth and proliferation [106]. Importantly, mTORC1 is integrated to the autophagic pathway via activation of TFEB and ULK1-ATG13-FIP200 (Family kinase-interacting protein of 200 kDa) complex [107,108]. Independent of mTORC1, AMPK activated the ULK1 complex under starvation [109]. Under nutrient rich conditions mTORC1 phosphorylated ULK1 and inhibited the ULK1-AMPK interaction to block autophagy [109]. It is important to consider that the TFEB, ULK1, and AMPK pathways are known to be regulated by amino acids, which contributes to another mode of autophagy regulation [110]. In a model of lung cancer, amino acid starvation led to an induction of non-selective macro-autophagy. However, amino acid starvation has been shown independent of mTORC1 to induce micro-autophagy that directly engulfs receptors of selective autophagy including NCOA4, LC3B, and SQSTM1 into endosomes [20]. The authors suggest these functions may prevent selective macro-autophagy and promote non-selective autophagy under starvation. Interestingly, under leucine starvation, a cleaved form of SQSTM1 is generated by the protease caspase-8. Under starvation when autophagy is active, a portion of the available SQSTM1 is cleaved. In nutrient replete conditions, this cleaved protein activates mTORC1 to increase leucine sensing [111]. The cleaved SQSTM1 is not able to participate in autophagy preventing opposing functions between mTORC1 and autophagy. Moreover, in ovarian cancer cells, arginine deprivation activated autophagy to promote cell survival [112]. Inhibition of autophagy both chemically or genetically significantly reduced cell growth. While these studies were not in CRC, these findings highlight the potential of combinatorial therapeutics with autophagy inhibitors and treatments such as arginase for tumors that rely on arginine for growth [112]. Glucose uptake plays a critical role in the growth of many cancer types, including CRC. In glucose-free conditions, knock-down of autophagy-associated genes increased cell death [113]. Similarly, when colon cancer spheroids were stressed under restricted glucose or serum an increase in autophagy was observed [114]. Under similar starvation conditions, *Kras* mutant tumors require autophagy for oxidative metabolism [115]. Starvation also affected expression of claudin 1 in colon cancer. Expression of claudin 1 was higher in tumor tissue and showed co-staining with lysosomal markers LAMP1 and 2 with increased autophagy. Under starvation, claudin 1 expression increased mediating a reduction in SQSTM1. This suppression suggests claudin 1 cross-talks with autophagy under starvation [116]. When and how nutrient availability impacts autophagy is essential in understanding its function in CRC tumors (Figure 3B).

### 4.2. Hypoxia

Hypoxia plays a key role in CRC development and progression. Hypoxia signaling is mediated by two transcription factors, hypoxia-inducible factor (HIF)-1α and HIF-2α, which have overlapping and distinct functions. In CRC, HIF-2α (not HIF-1α) is essential for CRC growth and progression [117]. Hypoxia is a well conserved cell stress that activates autophagy [43]. In tumor hypoxic foci, autophagy levels are highly elevated but rapidly subside upon establishment of a blood supply [118]. In colon cancer there is a known connection between hypoxia and mitophagy. Hypoxia disrupted mitochondrial respiration leading to increased mitophagy (Figure 2) [119]. Moreover, HIF-1α upregulated BNIP3 to induce mitophagy [43]. In patient derived CRC cells, inhibition of autophagy with 3-Methyladenin in combination with hypoxia, increased apoptotic death in cancer cells [120]. Moreover, the micro RNA miR-20a was found to inhibit hypoxia induced autophagy [121]. Additionally, in glioblastoma, HIF-1α induced autophagy and drove tumor growth [122]. HIF-1α does not alter CRC tumorigenesis in mouse models [117], however it will be interesting to assess if HIF-2α has overlapping roles in the context of autophagy.

### 4.3. Microbiota

As highlighted briefly above, autophagy can play an important role through xenophagy in managing the host–microbiome interaction. Moreover, dysregulation of autophagy is well characterized in IBD. New work studying chronic colitis suggests that autophagy protected cells by reducing apoptosis through upregulation of tumor necrosis factor-α [123]. As discussed above, the importance of autophagy specifically in Paneth cells is known [96]. In healthy tissue, induction of autophagy in Paneth cells induced interferon gamma to protect against microbiota. However, when this mechanism is lost, intestinal inflammation is exacerbated [124]. Consistent with data from IBD, the heterocellular cross-talk with microbiota is a major factor in tumor-elicited inflammation in CRC. When microbiome composition is altered under chronic inflammation or barrier defects, changes in the inflammatory response altered tumorigenesis [125,126]. The cross-talk between the microbiota and immune system highlights the complexity of the tumor microenvironment in the colon [127,128]. The importance of these mechanisms have been studied in depth [129,130]. Loss of autophagy in healthy colon epithelial cells through *Atg5* disruption altered the composition of the gut microbiota and the gut immune response suggesting implications in chronic colitis [131]. Similarly, loss of *Atg7* in intestinal epithelial cells and tumor tissue led to infiltration of anti-tumor immune cells decreasing tumor burden [75]. Treatment with antibiotics attenuated this response, further supporting a novel integration of microbiota and autophagy in tumor growth. It is important to highlight that this work utilized an *Apc* model where tumor suppressor p53 was intact. In many cancers, however, p53 is deleted or mutated thus these findings may only be applicable to patients with wild-type p53 [75]. In summary, the above findings highlight the cross-talk between the microbiota and autophagy. Further mechanistic studies may uncover novel therapeutic approaches targeting autophagy and microbiota.

## 5. Autophagic Substrates

The broad use of autophagy to meet metabolic demands is reviewed by Rabinowitz and White [132]. Autophagy in normal cell physiology is critical to maintain amino acid levels [133]. While it is thought that the products of autophagic degradation are recycled for use in cancer, in CRC the substrates targeted for autophagy and how the degradative products are utilized is not clear. In cancer, autophagy can degrade macromolecules for nutrients, and degrade tumor suppressors or oncogenes to alter growth. Below we outline both of these functions.

Previous literature has found an increase in autophagy in CRC spheroids under glucose or serum restriction [114]. However, the degradative products of this process are unknown. A study investigated this question by studying loss of *ATG5* in *RAS*-driven cancer cells. Loss of *ATG5* showed global changes in the proteome. Inhibition of autophagy, in combination with starvation, increased endoplasmic reticulum chaperones, proteins involved in DNA replication, and Rig-I like receptor signaling pathway. However, proteins that are known to be essential in stress survival were not altered with autophagy inhibition under starvation conditions [82]. This work uncovers how autophagy impacts cellular response to stresses such as starvation that are observed in the tumor microenvironment. Additionally, in *RAS*-driven cancers, autophagy drove glycolysis [83]. Degradation of cellular components into amino acids is essential for cancer utilization. Thomas and colleagues demonstrated that amino acid levels in starved breast cancer cells increased with activated autophagy, whereas normal cells maintained amino acid levels under starvation. It is hypothesized that this is due to the high nutrient demand to maintain the proliferation rates of the cancer cells [134]. While this study was not in CRC this underscores the importance of understanding how autophagy is used for nutrient acquisition.

While autophagy may be employed to acquire nutrients, it has been shown to break down proteins that activate or block tumor growth. Autophagy can cause the degradation of dishevelled in colon cancer and contribute to the activation of Wnt signaling, thus promoting tumor growth [135]. Similarly in CRC, the cancerous inhibitor of protein phosphatase 2a (CIP2A) is overexpressed [136]. CIP2a is involved in Myc protein stability. Temsirolimus, an FDA approved mTORC1 inhibitor that activates autophagy led to degradation of CIP2a and cell death in CRC [87]. In colon cancer, CyclinD1 is highly expressed and contributes to hyper-proliferation. Estrogen receptor beta was shown to activate autophagy and cause the breakdown of CyclinD1 causing cell cycle arrest and tumor death [88]. To prevent growth, treatment with 4-hydroxytamoxifen caused degradation of KRAS through autophagy in colon cancer [89]. As mentioned previously, basal autophagy breaks down FOXO3a to prevent apoptosis in CRC, to promote tumor growth [77]. While some work has been done, the process of breaking down proteins to inhibit or promote tumor growth are not well studied. A thorough understanding of autophagy in the context of CRC is important in targeting these mechanisms.

## 6. Conclusions and Future Perspectives

In general, non-selective autophagy is used for nutrient stress while selective autophagy is used for cell maintenance. However, in the context of tumor growth in CRC or CAC, these roles may change. Understanding the autophagic substrates that are recycled and how those substrates are utilized in tumor growth and development will identify ideal targets for treatments. While we have discussed mechanisms by which tumor cells may obtain nutrients through autophagy, these mechanisms are not clearly defined in CRC. The cross-talk between hypoxia and mitophagy underscores the importance of these mechanisms in CRC. Identifying the role of selective autophagy for tumor growth will allow the development of targeted therapeutics for CRC. The potential importance of mitophagy in cell stress and nutrient availability highlights a potential target in cancers. Moreover, if tumor cells employ selective autophagy for growth and survival, these mechanisms may be targets for vulnerability in CRC. Some of these approaches are reviewed by Martins and Baptista [137]. Additionally, we have highlighted the cell type specific contributions of autophagy and more precise work on cell type specific dependency on autophagy will shed light on the mechanistic role of autophagy in tumor development. More directly, the pathways activated or inhibited during nutrient stress and how autophagic substrates are being utilized in cancer cells will be critical to understanding the pleiotropic role of autophagy in cancer growth and progression.

## Figures and Tables

**Table 1:** Functions of autophagy in CRC. **A**. Observation/Autophagy indicates what mechanisms are observed in CRC tumors and if autophagy is active or inactive. Tumor response is a summary of whether or not the autophagy activity indicated generates a pro- or anti- tumor response. **B**. Summary of therapies and their modulation of autophagy. Treatment: Which therapy was employed. Autophagy: how the stated therapy modulated autophagy activation. Tumor response: how manipulation of autophagy via therapeutic treatment impacted tumor growth.

**A**				
**Observation**		**Autophagy**	**Tumor response**	**Reference**
**Activated chaperone-mediated autophagy in tumors**		Active	Pro-tumor	[19]
**Epithelial mitophagy increases CD8+T-Cells**		Active	Anti-tumor	[34]
**Loss of PARK2 accelerates tumor development**		Inactive	Pro-tumor	[38]
**Decreased ATG5 in CRC patients**		Inactive	Pro-tumor	[73]
**Increased ATG5 yields increased invasion**		Active	Pro-tumor	[73]
**Active autophagy through LC3B and SQSTM1**		Active	Pro-tumor	[74]
**Loss of ATG7**		Inactive	Anti-tumor	[75]
**RACK1 induces autophagy**		Active	Pro-tumor	[76]
**High Beclin-1 in CRC**		Active	Pro-tumor	[80]
**Increased LC3 with loss of p53**		Active	Anti-tumor	[81]
**Autophagy suppresses immune response in KRAS cancer**		Active	Pro-tumor	[82]
**Autophagy drives glycolysis in RAS cancers**		Active	Pro-tumor	[83]
**B**				
**Treatment**	**Autophagy**	**Tumor response**		**Reference**
**Mito-CP or Mito-Met10**	Active	Anti-Tumor; Decreased proliferation in KRAS mutant cancers.		[47]
**BH3 mimetic and chloroquine**	Inactive	Anti-Tumor; Induced apoptosis.		[48]
**Bafilomycin A1 or chloroquine**	Inactive	Anti-Tumor; Elevated FOXO3a and transcriptional upregulation of pro-apoptotic genes.		[77]
**Brevlin A**	Active	Anti-Tumor; Promoted expression of LC3-II and induced autophagy.		[79]
**KRAS siRNA**	Inactive	Anti-Tumor; Inhibiting mutant Kras inhibits autophagy and induces apoptosis.		[84]
**Vorinostat with chloroquine**	Inactive	Anti-Tumor; Induced apoptosis.		[85]
**5-Fuorouracil and chloroquine**	Inactive	Anti-Tumor; 5-FU treatment induced autophagy for resistance. Inhibition of autophagy reduced growth.		[86]
**Temsirolimus**	Active	Anti-Tumor; Inhibited mTOR to activate autophagy and degrade CIP2A.		[87]
**Estrogen Receptor Beta**	Active	Anti-Tumor; Autophagy directed CyclinD1 degradation inhibited growth.		[88]
**4-Hydroxytamoxifen**	Active	Anti-Tumor; Degradation of KRAS through autophagy induced cel death.		[89]
